# Insights from *Zootaxa *on potential trends in zoological taxonomic activity

**DOI:** 10.1186/1742-9994-8-5

**Published:** 2011-03-18

**Authors:** Elise Tancoigne, Cyprien Bole, Anne Sigogneau, Alain Dubois

**Affiliations:** 1 UMR 7205 OSEB, Département Systématique et Evolution, Muséum national d'Histoire naturelle, CP 30, 25 rue Cuvier, 75231 Paris Cedex 05, France; 2 SAP2S-DASTR, CNRS, 3 rue Michel Ange, 75794 Paris Cedex 16, France

## Abstract

**Background:**

An opinion currently shared by taxonomists and non taxonomists alike is that the work of inventorying biodiversity is unbalanced: firstly, in favour of countries in which taxonomy has been studied for a long time, and, secondly, in favour of vertebrates. In the current context of threats of species extinction, access for taxonomists to biological material and information becomes crucial if the scientific community really aims at a better knowledge of biological diversity before it is severely and irreversibly impoverished. We performed an analysis of 748 papers published in Zootaxa in 2006 and 2007, as well as 434 questionnaires sent to their authors to test these opinions. A generalization of these results to zoological taxonomy as a whole is discussed.

**Discussion:**

We found that the disequilibrium is not exactly what it usually considered to be. The USA, China and Brazil are currently the three leading countries in zoological taxonomy. Each of them presents, however, a different pattern. Taxonomists from Asia and South America are younger and mainly work in universities, not museums. A bias in favour of vertebrates still exists if we refer to the effort invested in each group to produce taxonomic data, but not to the number of papers. Finally, we insist on the idea that "describing a species" is very different from "knowing a species".

**Summary:**

The *taxonomic involvement *of a country, in terms of manpower and funding, appears to be a key factor in the development of fruitful taxonomic research. This message seems to have been understood by the countries that recently decided to increase considerably their taxonomic involvement. It still has to be received by those who did not.

## Background

Taxonomy is the disciplinary field of biology in charge of collecting, studying, describing, classifying and naming living organisms and taxa. It is a fundamental discipline for biology as a whole. It is thus crucial that taxonomists be given access to biological material and information. Previous studies [1-4] and widespread opinions [5,6] nevertheless postulate that access to specimens is unbalanced, both as regards the different regions of the world and the taxonomic groups. First, countries and areas where researchers have historically worked for a longer time on zoological taxonomy (Europe, North America and Australia) would therefore dominate zoological taxonomy. Second, the attention paid to Vertebrata would be higher than for other taxa, especially Arthropoda.

Yet, a quick search of all papers describing new species in the *Web of Science *in 2006 reveals that Brazil and China are among the top five publishing countries (USA: 25%, China: 13%, Germany: 8%, Japan: 7%, Brazil: 6%).

So, as concerns the distribution of zoological taxonomy, where are we now? No recent quantitative study exists at the world scale. Such surveys were either limited to a taxon [3,7-11], or a country [12-22], or they offered a summary of fragmented information [23-25].

However, today a mega-journal of taxonomy, *Zootaxa*, provides a worldwide coverage and deals with numerous zoological taxa. In 2004, it published far more pages than the combined total published in the ten core journals in systematic zoology [26]. It contributed 13% of all new taxa of animals indexed in the *Zoological Record *since 2004 [27]. An analysis of this journal could therefore produce a useful and relevant insight on today's taxonomical practices in zoology.

Quantitative tools will be used in this work to answer two questions:

1. Is zoological taxonomy still mostly practised in countries that have historically been taxonomically active?

2. Is zoological taxonomy still unbalanced in favour of vertebrates, *vs*. arthropods?

In conclusion, we will discuss the possible generalization of our results to taxonomy as a whole.

We determined the countries and areas in which (1) more works are published in zoological taxonomy; (2) most of the active taxonomists are working; (3) foreign taxonomists are mainly received; (4) taxonomists are involved in collaborations; (5) more species are described from other areas; (6) types specimens are kept; and, finally, (7) research is carried out in museums, which are traditional institutions for taxonomic research [9,15,28-30].

The disequilibrium in favour of vertebrates can be documented in terms of either taxonomic production (publications, pages, new species), or in the means employed for the studies (number of taxonomists, of specimens, of characters...). Therefore, we gathered additional information on (8) the taxa to which are devoted the higher number of publications, pages, new species as well as the taxa represented by the highest numbers of (9) taxonomists, (10) characters and (11) specimens used.

## Methods

### Sampling procedure

We chose to work on the years 2006 and 2007 because they were those when most articles had been produced at the time of our study (*n *= 2138). We randomly selected 748 articles among all those published in *Zootaxa *during this period. We devised a method to fix the sampling size. This method is explained below. Then we used three main procedures to obtain the data from our sample: data extraction from literature, data mining on databases and a questionnaire sent to each author of the sample who had provided an email. The data obtained from the articles or the questionnaire were entered into a database built using Access2002.

**Figure 1:**
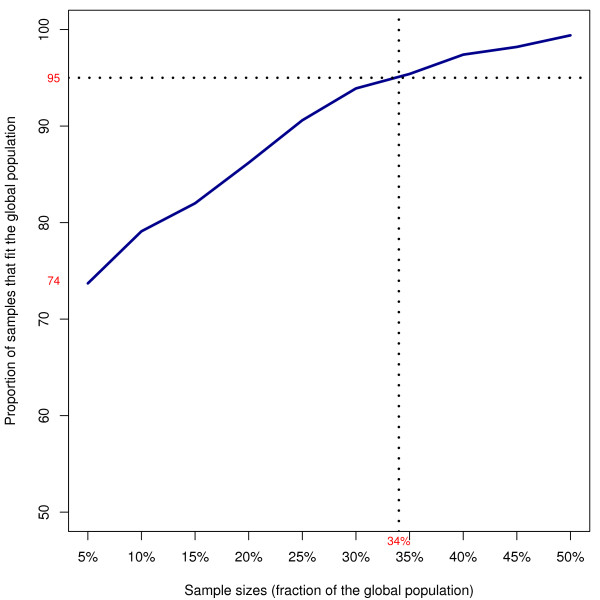
**Proportion of samples that correctly represents the total set of articles when the sample size is growing**. Eight parameters were tested both over the total set and over the samples. The probability of drawing a correct sample is 95% when the sampling size is 34% of the global population.

Five parameters describing each publication of the whole set of 2138 articles published in *Zootaxa *in 2006-2007 were available in the *Zoological Record online*: (1) treated taxon, (2) number of pages, (3) number of authors, (4) number of described species and (5) number of references. Three other parameters found on the *Zootaxa *website [31] were added: (6) whether the article is in open access or not, (7) its delay of publication after submission and (8) its category (article or monograph). A sampling script was written with the R language [32]. We made 1000 random samplings of 5% of the total number of papers (function *sample() *without replacement of the default package *base*). This operation was repeatead nine successive times with increasing sample sizes. Thus, we obtained 1000 samplings of 107 articles, 1000 samplings of 214 articles, etc. Then we used statistical tests to determine the rate of correct samples that each operation produced. Each of the 10,000 samples was tested and considered correct if the eight parameters did not significantly differ from the whole set (*P *< 0.05). As our data did not verify the normality condition, non-parametrical tests were performed for each of the eight parameters: χ^2 ^for (1); Wilcoxon/Mann-Whitney for (2), (3), (4), (5), (7); and binomial tests for (6) and (8). We decided to choose the minimal sample size for which 950 out of 1000 samples would not statistically differ from the whole set. This size is 35%, i.e., 748 articles (Figure 1). Finally, a random sampling of 748 articles was performed. We also checked that it did not significantly differ from the whole set for each of the eight parameters.

### Questionnaire

The 748 articles studied were written by 1778 authors. Once multiple authorships were removed, 1384 authors remained, 1025 of whom had mentioned an e-mail address in the paper. A questionnaire had been sent via e-mail in May 2008 to 10% of these 1025 authors for a preliminary study. It received 56 answers and comments from authors from most parts of the world (except Africa). Our questionnaire was considered approved and it was then sent once again in May 2009 to the 939 remaining authors. It was rejected by 105 mailboxes and received 434 answers in all (56 in 2008 and 378 in 2009). Its answer rate was thus 47%. We used EmailQuestionnaire software to send our questionnaire [33].

### Data picking

We obtained data for the 748 sampled articles; the 1778 authors and their 1111 institutions; the 1413 new species that were described in it and their 1404 holotypes; 15 syntypes; 23 468 paratypes and the 4987 other specimens used in descriptions. The 1778 taxonomic authors corresponded to 1384 "real persons", of whom 434 taxonomists sent us personal data. Table 1 presents the data.

**Table 1:** Data extracted for each article, author, institution, new species and specimen involved

Article		
**ZR ID number**	Number attributed by the *Zoological Record*	ZR

**Title**		ZR

**Abstract**		ZR

**Internet link**	Internet link to the pdf article on the *Zootaxa *website	ZT

**Number of pages**	*n *= (ending page - beginning page) + 1	ZR + H

**Delay of publication**	Number of days between acceptance and publication	ZT + H

**Category of document**	*Monograph*; *article*	ZT

**Category of work**	We distinguished six categories of works. *additional data*: addition of sounds, karyotypes, redescriptions, chorology, phylogenies to the knowledge of a taxon, without new taxon description; *checklists or catalogue of species/types*; *regional fauna*: taxonomic revision at regional scale; *taxonomic revision*: each taxon or types reviewed, or at least 3 different kinds of analyses done (e.g. morphological studies plus distribution plus karyotype of a taxon); *taxonomic work on isolated taxa*: single or multiple descriptions of new taxa, new descriptions plus new records of the taxon; *theoretical works*: biographies or nomenclatural changes only, or theoretical work on taxonomy and nomenclature	H

**Authorship presentation**	Whether authorship is in alphabetical order or not. Names beginning by "De", "Van", are considered at letters "D" or "V".	H

**Phylum delimitation**	Phylum as provided by ZR indexation	ZR

**Taxon delimitation**	The most inclusive taxon treated in the article	H

**Level of work**	Level of the most inclusive taxon treated in the article. *Species level*: any level up to and including species level; *genus level*: any level from species level up to and including genus level; *family level*: any level from genus level up to and including family level; *above family level*: any level above family level	H

**Geographic delimitation**	The most single inclusive area treated in the article. We used political boundaries of studied areas instead of biogeographical ones, in order to study the relationship between laboratories and studied areas	H

**Level of geographic delimitation**	We used four levels of area: *locality level*, *regional level*, *country level*, *major area level*. We used *other *when the study involves more than one major area. Oceans and seas were treated as regions and affiliated to a country or a continent when specified in the article	

**Number of references**		ZR

**Price**	Whether the article is in free access or not	ZT

**Authors**		

**Family name**		ZR

**Firstname**		ZR

**Article related**	List of the articles written by the author in *Zootaxa*	H

**Position in the article**		H

**Institution**	Institution of the author for the paper concerned. The ZR provides the address of the first co-author; the other ones were added from the paper itself. Whenever the author is affiliated to several institutions, this information was retained	ZR + H

**Email**	Some email addresses were provided by the ZR; others were found in the article	ZR + H

**Professional status**	*Amateur, Doctoral student, Established taxonomist researcher, Established non-taxonomist researcher, Master student, Postdoctoral, Retired researcher, Technician*	Q

**Nationality**	Country of birth	Q

**Age**		Q

**Sex**		Q

**Highest degree**	*Master (four or five years of faculty)*, *PhD (or equivalent)*, *Other*, *No degree*	Q

**Free comment**		Q

**Institutions**		

**Name**	The names of all institutions were checked to suppress duplicates	ZR + H

**Type**	Five categories of institutions were recognized: *Museum, University, Institute, Private, Other institution*	H

**Country**		ZR + H

**Continent**	The affiliation of a country to a major area followed the CIA World Factbook data [34]	H

**Level of the institution**	Some institutions are subordinate to others, which may (or may not) appear in the address given by the author(s). For each of them, the following information was checked using their web sites: 1: whether the institution is autonomous 2: whether it is subordinate to another institution 3: whether it is subordinate to two other institutions	H

**Subordinating institution**	Name of the superordinate institution if relevant	H

**New species**		

**Nomen**		ZR

**Specimens involved**	List of the specimens involved	H

**Allotypes**	Whether allotypes are designated or not	H

**Developmental stages**	Whether numerous developmental stages are involved or not	H

**Methods used for tree construction**	*Parsimony, Bayesian/maximum likelihood, Distance, Combined methods*	H

**Characters used**	*Morpho-anatomical, Molecular, Bio-acoustic, Karyological, Other*	H

**Specimens used to describe a new species**		

**Number of specimens**	Number of specimens of each type for each new species	H

**Kind of specimen**	*Holotype*, *Syntype*, *Neotype*, *Paratype*, *Other specimen*	H

**Place of collect**	Collecting locality as stated in the paper, with GPS coordinates if available. Localities were distinguished through their names or through their GPS coordinates. Each locality was referred to a country and a realm whenever possible. The geographical delimitations used are the same as for the publications	H

**Institution of conservation**	Institution where specimen(s) is/are kept	H

**Year of collect**		H

**Size (cm)**	For holotypes only	H

**Sex**	For holotypes only. *Male*; *Female*; *Hermaphrodite*; *Unknown *when the information is lacking. Because many authors do not indicate the sex when they use juveniles, we also added the category *Juvenile*	H

H: data added by hand. Q: data from the questionnaire. ZR: data from the *Zoological Record *online. ZT: data from the *Zootaxa *website.

Each country was affiliated to a region through the data of the CIA world factbook [34]. Major regions are abbreviated the following way: Af (Africa), As (Asia), E (Europe), M (Middle East), N (North America), O (Oceania), S (South America). Given the small amount of papers from Central America, this region was grouped with South America.

Authors (i.e. names on paper) and taxonomists (i.e. real persons) were distinguished. For example, a taxonomist who writes two articles represents two authors.

### Analyses

The number of publications per major region or country was computed fractionally. For example, an article written by two authors from Asia and one author who mentioned two addresses, one in South America and one in Africa, counts for 0.5 for Asia, 0.25 for South America and 0.25 for Africa. The number of publications per category of institution was also calculated fractionally. For example, an article written by two authors from the University of Sao Paulo and one author who mentioned two addresses, one in the University of Sao Paulo and one in the American National Museum, counts for 0.75 for Brazilian universities and 0.25 for North American museums. The number of type-specimens per category of institution is calculated directly. For example, a new species with one holotype kept in the University of Sao Paulo and 54 paratypes kept in the American National Museum counts 1 for North American museums and 54 for Brazilian universities. The number of taxonomists per major region or country was calculated fractionally once duplicated authors (authors with same name and address) had been removed. For example, if an author mentioned two addresses in two different major regions s/he counts 0.5 for each region. The collaboration indicator of the *Observatoire des Sciences et des Techniques *(Paris, France) was used to study collaborations: (number of publications of a region A with an other region B)/(total number of publications of region A). For instance, an article written by one author from Asia and two from South America counts for one co-publication between Asia and South America.

All the data was analysed with the free statistical software R [32] and packages *ade4 *[35], *RODBC *[36], *vcd *[37,38] and *FactoMineR *[39]. We used correspondence analysis (CA) to summarize the data's structure and identify similarities between the variables of a contingency table [40]. CA measures the distances between the row and column points and presents the inter-relations of variables in calculated dimensions that outlines maximum of variance (VARIMAX rotation). The analysis determines which category values lie close together and which are far from each other. These can be seen on a correspondence map, where the row and column categories are plotted along the computed factor axes. Spatial proximity between categories does not imply a correlation between the categories but instead a strong specific link in relation to these factors. This graphical method is based on a χ^2 ^test that measures the deviation to independence model. We also performed χ^2 ^statistical tests to compare proportions when it was needed.

### Evaluation of *Zootaxa *biases

Our choice to work on *Zootaxa *inevitably represents a bias. Because our questions treat the different areas of the world and taxa, two possible biases concern the representativeness of (1) the taxa studied in *Zootaxa *and (2) the regions where the studies were carried out.

**Figure 2:**
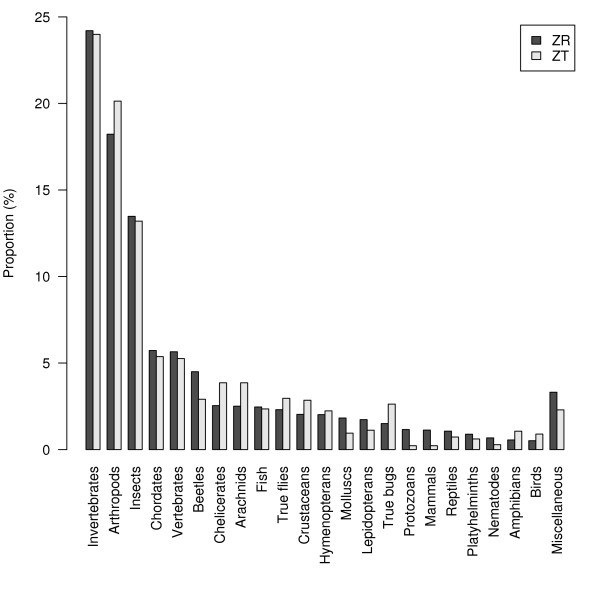
**Comparison of the proportion of taxa studied in all works publishing new species in 2006 and indexed in the *Zoological Record *(ZR, 5620 articles) and in the *Zootaxa *subset (ZT, 743 articles)**. Search query: expression "sp nov" from ZR thesaurus AND "2006".

We compared the proportion of the different taxa studied in 2006 in *Zootaxa *and in all the publications indexed in the entire volume of the *Zoological Record *for 2006 (Figure 2). We observed that "taxo*", "taxonomy" or "systematic" did not even allow us to find all articles published in *Zootaxa *in 2006. We used the keyword "sp nov", coming from the *Zoological Record*'s thesaurus, to find these publications in the *Zoological Record*. If all categories of Figure 2 are considered, a χ^2 ^test shows significant differences (*P *< 0.001) between the *Zoological Record *and *Zootaxa*. However, the distribution does not show significant differences (*P *= 0.12) if we consider only three classes of taxa: 'Arthropods', 'Vertebrates' and 'Other'. Therefore, for the purposes of our study the taxa were grouped as 'Vertebrates', 'Arthropods' and 'Other'.

**Figure 3:**
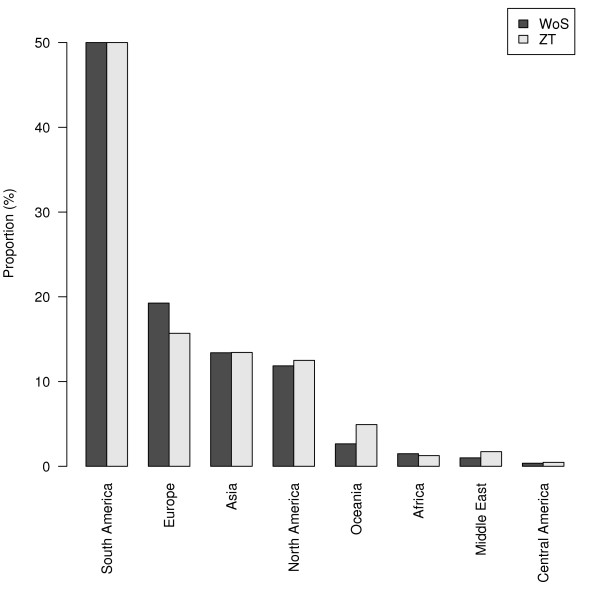
**Comparison of the proportion of regions publishing new species in 2006 in journals indexed in the *Web of Science *(WoS, 4313 articles) and in the *Zootaxa *subset (ZT, 675 articles)**. Search query: ("sp nov" OR "new species") AND "2006".

We also compared the proportions of the different regions where new species were described in 2006 in *Zootaxa *and in all the publications with the same pattern indexed in the entire volume of the *Web of Science *(WoS) for 2006 (Figure 3). We used the *Analyze Results *tool of the *Web of Science *which computes information on authors' countries. Our search query "'sp nov' OR 'new species'" found 675 articles for *Zootaxa *and 4313 articles for the *Web of Science*. We did not use the truncated query "new sp" because it returned articles about language ("speech", "spoken"). If all the regions of Figure 3 are considered, a χ^2 ^test shows significant differences (*P *< 0.001) between the *Web of Science *and *Zootaxa*. However, the distribution does not show significant differences if we consider only three classes of regions: Asia-South America (AsS), Europe-North America-Oceania (ENO) and Africa-Middle East (AfM) (*P *= 0.46); or Africa-Europe-Oceania (AfEO), South America-North America (SN) and Asia-Middle East (AsM) (*P *= 0.36).

## Results

Publications dealing with species are predominant in *Zootaxa *(74% of the works analysed). Only 17% of the papers deal with taxa between genus and family, and 9% with taxa above family level. Therefore, so-called "α-taxonomy", "microtaxonomy" [41], or "eidonomy" [42] is predominant. Works on isolated taxa (single species descriptions, etc.) are the most numerous (57%), followed by taxonomic revisions (17%), additions of new data for taxa already known (11%), regional faunas (8%), checklists or catalogues of species/types (4%) and theoretical works (3%). These terms are defined in Table 1.

**Figure 4:**
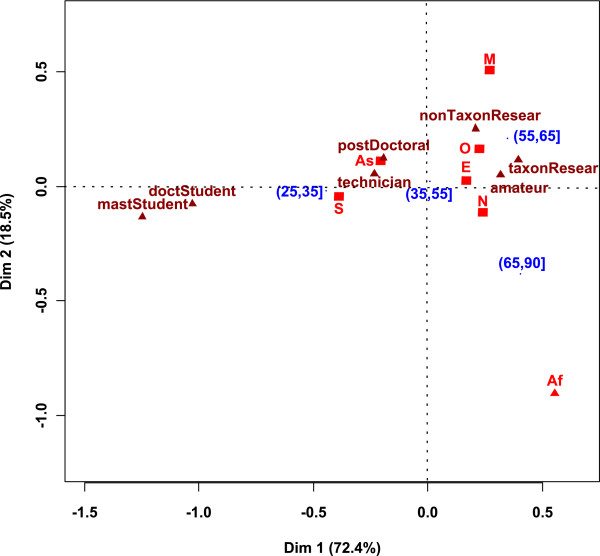
**Result of the CA between the authors' nationality and age**. *Af: Africa, As: Asia, E: Europe, M: Middle East, O: Oceania, N: North America, S: South America*. Professional status is used as supplementary data.

Most of the people who answered our questionnaire are fully qualified researchers (60%), either taxonomists (45% of total) or not (15%). Students come in second position, with 15% PhD students and 7% MSc students. Postdoctoral students account for 9% and retired researchers, amateurs and technicians for the remaining 9%. The proportions listed above vary between the three areas previously defined (Figure 4). Figure 4 shows the result of the CA between the age and the region of origin of the authors. Professional status was used as supplementary data. We deleted the 'retired researcher' category because it was naturally linked with the (65, 90] category and made the map confusing. Africa is distant on the map because it has few values (0, 2, 1, and 0) which produce high frequencies (0, 66, 33 and 0). Dimension 1 is strongly linked with the age and pits the (25, 35] category against the (55, 65] and (65, 90] categories. This is very interesting because this separation depends solely on the nationality categories. Indeed, (25, 35] is linked to S and appear to be the category with the highest proportion of S and the (55, 65] and (65, 90] categories are linked to M, O, N and appear to be the categories with the highest proportion of them. Being at the center of the map, the (35, 55] category represents the average category as related to nationality. E represents 38% of (35, 55], S 23%, N 16%, As 15%, O 4%, M 2%, Af 1%.

Dimension 2 separates M from the other categories because no individuals belonging to the (65, 90] category answered our questionnaire. The supplementary data show that the (25, 35] category was related to technicians and postdocs, whereas (55, 65] was related to established taxonomic researchers, established non-taxonomic researchers and amateurs. These data suggest that the population of taxonomists is younger in As and S.

Let us now consider the twelve questions that we posed above.

### Which areas and countries publish more in zoological taxonomy?

The region that provided the highest number of publications is Europe, and then come, grouped together, North America, Asia and South America (Table 2-A). In Europe, no country provided more than 20% of publications, the most productive country being Germany (20%). In contrast, for the other main regions, a single country usually accounts for more than half the total publications: the USA for most publications from North America (76%), China for most publications from Asia (52%), Brazil for most publications from South America (62%), South Africa for most publications from Africa (39%) and Turkey for most publications from Middle East (67%).

**Table 2:** Relationships between studied regions and regions of researchers' labs

	A Studying regions (%)	B Studied regions (%)
Asia	20	**25**

South America	21	**25**

Multi-areas	-	12

North America	21	8

Oceania	7	8

Europe	**29**	7

Africa	2	6

Middle East	2	3

Central America	1	3

Antarctica	-	1

Without region	-	2

Studying regions: percentage of a fractional count of authors of publications. Studied regions: percentage of the number of publications. "Multi-areas" refers to work performed on more than one continent. Asia and South America are the most studied areas and belong to the areas with the highest numbers of publications.

### In which areas and countries are taxonomists working?

The number of publishing taxonomists per country is the highest for Europe (27%), then for South America (24%), North America (21%) and Asia (20%). Germany is the first country of Europe (20%); Brazil is the first country of South America (61%), the USA of North America (79%) and China of Asia (51%).

### Which areas and countries mainly receive foreign taxonomists?

Concerning geographic mobility, 10% of the 427 authors who answered the question were not working in their country of nationality. Most were fully qualified researchers (69%), followed by predoctoral students (29%) and postdoctoral students (9%). Europeans are those who move the most (53%). Europe and North America are the areas with the highest rates of authors originating from other areas (37% and 25%, respectively). Unfortunately, our results on taxonomists' mobility do not allow us to know whether the weak mobility of taxonomists from Asia and South America to Europe, North America and Oceania is due to short-term mobility (which cannot be measured from our data) or to a more general lack of mobility.

### How are international collaborations structured?

**Figure 5:**
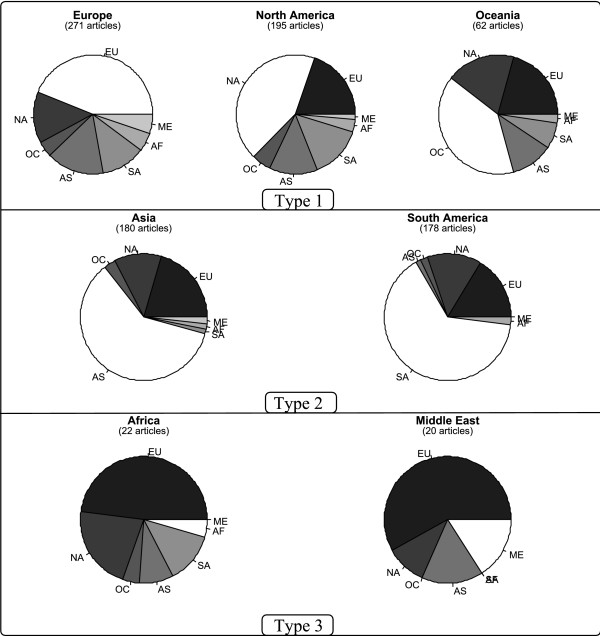
**Nature of the cooperation between researchers from different areas of the world, for articles whose number of authors exceeds 1**. We used the OST (Observatoire des Sciences et des Techniques, Paris, France) indicator: (number of publications of a region A with an other region B)/(total number of publications of region A).

Collaborations are numerous: 73% of the publications have at least two authors. Three kinds of collaboration patterns were observed, corresponding to our previous grouping in areas (Figure 5): regions which co-publish with every other area of the world (ENO); regions which mainly co-publish with themselves (AsS) or with ENO; and regions which do not publish much (AfM).

Authors from ENO area do not predominantly sign the publications as first authors (χ^2 ^test, *P *= 0.054).

The percentages of single-author articles is greater in ENO (28%) than in AsS (12%; χ^2 ^test, *P *= 9.49e-08).

### In which external areas and countries are new species described?

From a zootaxonomic point of view, Asia and South America are the most studied regions (see Table 2-B). Altogether they account for 50% of the publications and 61% of the new species. Latitudes are provided in the publications for 667 of the new species. Most of them (77%) come from latitudes higher than 10°, equally distributed between North and South. The remaining 23% are also equally distributed North and South (χ^2^: *P *> 0.05).

**Figure 6:**
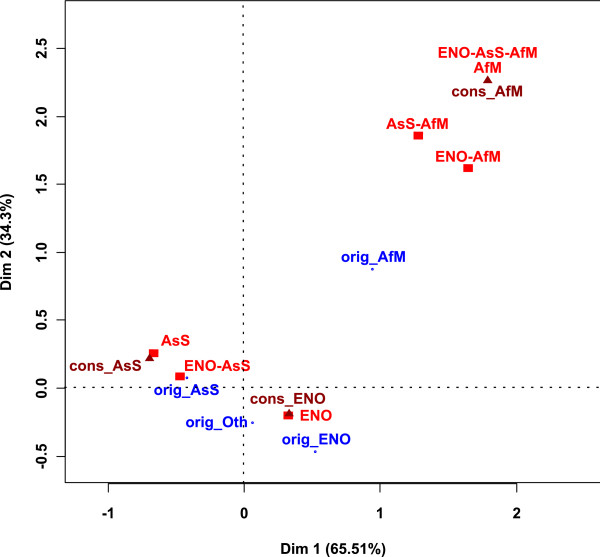
**Result of the CA between the region of origin of the holotype and authors laboratory**. The place of conservation of the holotype is used as supplementary data. *cons_: region of conservation of the holotypes; orig_: origin of the holotypes*. *Af: Africa, As: Asia, E: Europe, M: Middle East, O: Oceania, N: North America, S: South America*.

Figure 6 shows the result of the CA between the region of origin of the holotype and authors laboratories. The place of conservation of the holotype was used as supplementary data. Dimension 1 and dimension 2 together are strongly linked with both the origin of the holotypes and authors laboratories. They separate three groups: holotypes from AsS, holotypes from ENO and holotypes from AfM. AsS holotypes are linked to AsS collaborations and ENO-AsS collaborations whereas AfM holotypes are close to AfM, ENO-AfM, AsS-AM and ENO-AsS-AfM collaborations. However, AsS holotypes appear to be the category which is mainly studied by AS and ENO-AsS collaborations and AfM holotypes appear to be the category which is mainly studied by all areas of the world. The ENO holotypes category is linked to ENO collaborations and ENO holotypes appear to be studied by authors from ENO areas only. Thus, only ENO authors work on all areas of the world. Dimension 2 is linked with the atypical work on AM holotypes: authors from AfM are the only ones to work on their regions only, with numerous collaborations from all over the world.

### Which areas and countries house the holotype specimens?

The supplementary data of Figure 6 show that holotypes are not always kept in the areas they come from. Authors from ENO area keep their holotypes as well as the holotypes from Africa and Middle East which they have studied in collaboration. On the other hand, authors from Asia and South America keep their holotypes even if studied in collaboration with authors from Europe, North America or Oceania.

### Are museums preferred for carrying out taxonomic research and keeping the type specimens?

**Figure 7:**
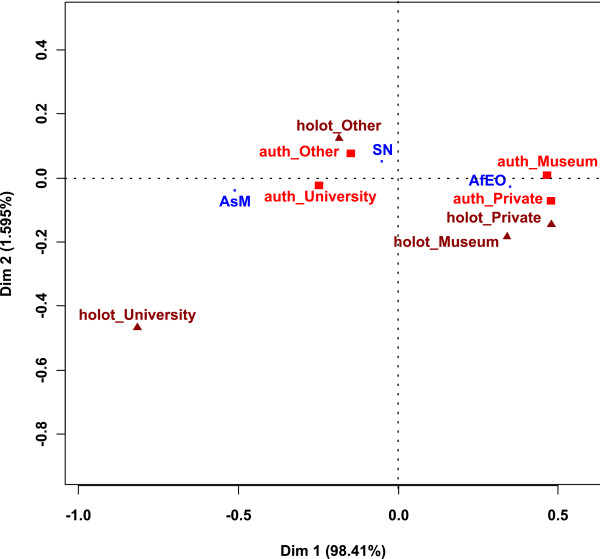
**Result of the CA between the geographical areas of authors and their type of institution**. The institution of conservation of the holotypes is used as supplementary data. *holot_: holotypes' type of institution; auth_: authors' type of institution*. *Af: Africa, As: Asia, E: Europe, M: Middle East, O: Oceania, N: North America, S: South America*.

Author affiliations to universities are numerous (47%), followed by museums (28%), other institutions (21%) and private addresses (4%). Figure 7 shows the result of the CA between the geographical areas of authors and their type of institution. The places of conservation of the holotypes were used as supplementary data. Dimension 1 is strongly linked with geographical areas and separates the AsM area from the AfEO area. Universities and other institutions of work are linked to AsM and SN, whereas Private addresses and Museums are linked to AfEO. The supplementary data show that housing institutions differ according to the region and follow authors' affiliations, except as concerns Universities. Holot_University is distant from auth_University on the map because a high number of museums are subordinated to universities (63% in our data), especially in AsM, and are thus not mentioned in the authors' address.

The proportion of museums in authors' affiliation decreases to 33% (AfEO), 3% (AsM) and 7% (SN) when the institution that subordinates the museum is taken into account. The proportion of universities also increases to 44% for AfEO, 70% for AsM and 67% for SN.

Noteworthy is the high proportion of museums (55%) and private collections (21%) for AfEO. 28% of the types kept in European private collections are holotypes and syntypes; 72% are paratypes.

### Are there more works on Vertebrata?

Most of the works on taxa (Table 3) deal with Arthropoda (69%), Chordata (16%), followed by Mollusca (3%) and Platyhelminthes (2%). Other taxa occur for less than 2% each and form the 10% remaining group. Arthropods are mainly Insecta (64%) and Arachnida (14%), whereas Chordata are only represented by vertebrates.

**Table 3:** Proportion of publication and new species for each taxon

	% of publication	% of new species
**Arthropoda**	**69**	**81**

Insecta	45	58

Arachnida	10	11

Malacostraca	8	9

Other	6	3

**Vertebrata**	**16**	**8**

Amphibia	5	3

Actinopterygii	4	2

Reptilia	4	1

Chondrichthyes	1	1

Other	2	1

**Mollusca**	**3**	**3**

**Platyhelminthes**	**2**	**2**

**Other**	**10**	**6**

*Sum*	*100*	*100*

Left: n = 735. Right: n = 1416.

It is thus not surprising that newly described species are mainly arthropods (81%) and vertebrates (8%). Many (51%) of the new species are described in taxonomic revisions, and only 35% in isolated works.

**Figure 8:**
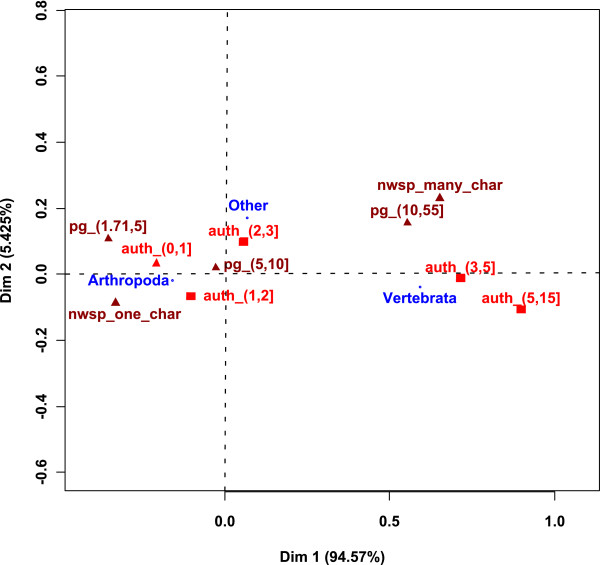
**Result of the CA between studied phyla and the number of authors of each document**. Supplementary data: number of pages per new species, diversity of characters used to describe each new species. *auth_: number of authors; pg_: number of pages per new species*.

Figure 8 shows the result of the CA between studied phyla and the number of pages per new described species of each publication. The diversity of characters used to describe each new species and the number of authors of each document are used as supplementary data. Dimension 1 is strongly linked with phyla and separates the Vertebrata from the Arthropoda. Vertebrata is linked to category (10, 55] whereas Arthropoda is linked to categories with fewer pages per new species.

### Are there more taxonomists involved in Vertebrata studies?

The supplementary data of Figure 8 show that the category Arthropoda is linked to few authors categories, whereas Vertebrata is linked to categories with more than three authors.

In the Vertebrata, a new species is described on average by 3.4 authors, whereas the figure is 0.9 in the Arthropoda. Thus, for an equal number of taxonomists, taxonomists working on arthropods describe more new species than taxonomists working on vertebrates.

### Are there more characters involved in Vertebrata studies?

The supplementary data of Figure 8 show that the variety of characters used to describe the new species is higher for vertebrates. Whenever non-morphological characters are used in Vertebrata descriptions, these are mainly molecular or bio-acoustic.

### Are there more specimens involved in Vertebrata studies?

No significant difference between taxa was found in this respect. One species out of five was described from one specimen only (20%), or known from one locality only (21%). As much as 53% of the new species were collected from more than 2 localities. Two species out of five were described from specimen(s) of one sex only. Nine species out of 10 were described on the basis of a single stage of development (no significant difference between taxa). These figures vary when the year of collection is taken into account. New species known only from 1 specimen represent 64% of the new species with holotypes collected before the median year 1999. They represent 33% of the new species with holotypes collected after 1999 (*n *= 1261, *P *= 5.83e-08).

## Discussion

### Is zoological taxonomy still more developed in countries that have been historically taxonomically active?

This study of *Zootaxa*'s 2006-2007 publications supports the idea that zoological taxonomy is still more developed in countries that have historically been taxonomically active. Taxonomists from Europe, North America and Oceania are the only ones to collaborate largely with those from other regions, and they do so with all regions in the world. They also remain the only ones to work on very wide geographical zones and to host researchers from abroad. A lack of studies in Africa has already been pointed out [11,25,43]. This lack still exists, as African regions are not well represented in our sample.

However, this study also shows that Asian and South American countries appear to play a prominent role in zoological taxonomy today. Their high number of publications, the fact that they keep their own specimens and the median age of their respondents show that they have a developed and active community of taxonomists. Previous studies on taxonomists' age could not be used for a rigorous comparison because the enquiries were not carried out at the same time [15,20,23,28,44-46].

Are these results artefacts created by our choice of the journal *Zootaxa*? We do not think so, for several reasons. First, we previously checked that our sampling was not biased questioning this respect. Second, the taxonomic growth of the two main countries responsible for these results (China and Brazil) has already been pointed out in the literature [10,47]. China's funding for research increased by 30% each year between 1998 and 2006, which benefited taxonomy: "*The evaluation of the funded projects in the Division *[of Zoology] *shows that animal morphology and taxonomy are the main supported areas, accounting for half of the total funded*" [48]. In 2004, China became the fifth country in terms of number of publications. The number of students has been increasing everywhere in this country [49]. An analysis of Brazil's scientific production carried out by The Observatoire des Sciences et Techniques (Paris, France) showed that it was quickly growing [50,51]. The figures were even underestimated: a bibliometric study [50] showed that many Brazilian articles are published in Portuguese, in national journals. Thus they were not included in the calculation. Moreover, a recent study on amphibian taxonomic effort and expertise supports these results concerning China and Brazil [52]. It also found that the continental distribution of authors in *Zootaxa *(*n *= 5663) was similar to the continental distribution of authors working on a given taxon (i.e. Amphibians, *n *= 647).

Another argument in favour of this idea involves the geographical origin of the new species. Surprisingly, no latitude preference was identified regarding the origin of the newly described species. This contradicts the widespread idea that latitudes close to the equator have a richer biodiversity [53]. Why, then, do our data fail to find high proportion of new species in the belt of 10° above and below the equator?

In this respect we found that places where the highest numbers of species were described recently (China, Brazil) are also those with the highest number of taxonomists. A correlation exists between the number of taxonomists and the number of species known in a phylum [54]. It is thus likely that the high number of newly described species in China and Brazil is a reflection of the *taxonomic involvement *of these countries, in terms of manpower and funding. Thus, if China and Brazil appear to be the most studied regions, this is not only because they are large countries with a rich biodiversity [55-57], but also because they have numerous taxonomists and have decided to invest in this branch of research.

Finally, it is striking that a high proportion of our respondents were professionals (fully qualified researchers, students) despite the high number of amateurs in entomology [9]. *Zootaxa *would therefore reflect a biased sub-sample of the taxonomic community, including mostly professionals. One may wonder whether part of this phenomenon might be due to the fact that some amateur taxonomists, especially in entomology, tend to publish their works in their national languages in national periodicals whenever they still exist [58]. Unfortunately, professional categories are not expressed the same way between studies, hence our figures are hardly comparable with previous works [15,24,28,54]. We also have to keep in mind that the authors who provided their email addresses are mainly the corresponding authors - a factor that might influence our results.

### Is zoological taxonomy still in favour of vertebrates, vs. arthropods?

On the basis of taxonomic output (number of publications, number of new species), arthropods, especially insects, appear to be much more studied than vertebrates. Vertebrates nevertheless concentrate a higher number of taxonomists, characters and methods used for each new species. Thus, even if more results are produced on arthropods, their study involves a lesser amount of means (number of taxonomists, number and diversity of methods used).

The long-studied vertebrates [2,59] are nowadays studied with modern methods. Most of the time, these methods complement the morphological ones. As a consequence, morphological studies are a necessary step in any taxonomic work. They are not a sign of intellectual or technological backwardness when presented alone [60]. They are a strong indication of the amplitude of our ignorance of the taxon in question. The data we gathered on the numbers of specimens, localities and kinds of characters used to recognize and describe a new taxon clearly show that we are very far from "knowing" a species once it has been "described" and named [61].

Moreover, the taxa which benefit from a high taxonomic involvement have nevertheless the lowest rates of description of new species. If we aim at increasing the number of known species as quickly as possible, taxonomists should give priority to taxa which do not require these new methods.

We do not think that these results are artefacts created by our choice to work on the journal *Zootaxa*. We previously checked that our sampling was not biased for this question and we already know that vertebrates have been studied for a longer time [2,59]. Our observations match this idea.

This disequilibrium of the taxonomic effort in favour of vertebrates is problematic if we aim at better knowing that which we know the least. Insects represent 58% of the new described species and vertebrates 7.2%. Yet, according to some estimates (figures computed from the sum of the 'World Descr.' and 'Estimate World' figures for chordates and invertebrates in [56]), vertebrates would only represent 0.3% of the numbers of species that remain to be described, whereas insects represent 74%. Thus, this distribution of taxonomic effort between taxa is unsuitable when considering the needs [16,23,24,54,62]. The international and national scientific communities should hire more people to focus on arthropods, especially entomologists.

The nature of the works published in *Zootaxa *show that this journal gives priority to alpha-taxonomy. "Zootaxa *considers papers on all animal taxa, both living and fossil, and especially encourages descriptions of new taxa*" [63]. We therefore consider our main results valid for zoological alpha-taxonomy.

## Conclusions

We found that the disequilibrium does not correspond exactly to that usually admitted. The USA, China and Brazil are currently the three leading countries in zoological alpha-taxonomy. However, each of them presents a different pattern. Taxonomists from Asia and South America are younger and mainly work in universities, rather than museums. A bias in favour of vertebrates still exists if we refer to the effort invested in each group to produce taxonomic data, but not for the number of papers. Finally, we insist on the idea that "describing a species" is very different from "knowing a species". The taxonomic involvement of a country, in terms of manpower and funding, appears here to be a key factor in the development of fruitful taxonomic research. If we aim at increasing our knowledge of the species of the world, we still have much efforts to make in terms of the efficient distribution of the taxonomic effort between taxa.

## Competing interests

The authors declare that they have no competing interests.

## Authors' contributions

ET designed the sampling procedure and the database, compiled the database, participated in the statistical analysis and drafted the manuscript. CB designed and participated in the statistical analysis. AS participated in the analyses of the results and helped to draft the manuscript. AD designed the study, participated in the analysis of the results, and helped to draft and translate the manuscript. All authors read and approved the final manuscript.
